# The correlation between the lumbar disc MRI high-intensity zone and discogenic low back pain: a systematic review and meta-analysis

**DOI:** 10.1186/s13018-023-04187-5

**Published:** 2023-10-07

**Authors:** Lei Yang, Wenhao Li, Yongdong Yang, He Zhao, Xing Yu

**Affiliations:** 1 Department of Acupuncture and Moxibustion, Dongzhimen Hospital, Beijing University of Chinese Medicine, Beijing, 100700 China; 2 Department of Orthopedics III, Dongzhimen Hospital, Beijing University of Chinese Medicine, No. 5 Haiyun Warehouse, Dongcheng District, Beijing, 100700 China

**Keywords:** MRI, High-intensity zone, Discography, Discogenic low back pain, Meta-analysis

## Abstract

**Objective:**

This study aimed to investigate the correlation between the MRI high-intensity zone (HIZ) and the pathogenesis of discogenic low back pain.

**Methods:**

Literature from PubMed, EMBASE, Cochrane Library, Science Direct, China Knowledge Network, Wanfang Database, and China Biomedical Literature Database was searched until August 2023. Cohort studies including patients with low back pain who underwent lumbar spine MRI and discography, as well as the results evaluating the correlation between HIZ and discography for morphological changes in the disc and pain replication phenomena, were included in the analysis. The literature that met the inclusion criteria was screened, and the methodological quality of the included studies was evaluated. Meta-analysis of the extracted data was performed by using RevMan 5.1.1.

**Results:**

In total, 28 reports were included in this meta-analysis. There was a statistically significant correlation between a positive HIZ and abnormal disc morphology in discography (OR 28.15, 95% CI [7.38, 107.46], *p* < 0.00001). Patients with HIZ-positive discs had a significantly higher incidence of consistent pain (71.0%, 969/1365) than those with HIZ-negative imaging (29.0%, 1314/4524) (OR 7.71, 95% CI [5.29, 11.23], *p* < 0.00001).Segments that were HIZ-positive and had abnormal disc morphology had a higher incidence of consistent pain (86.1%, 230/267) than HIZ-negative subjects (32.2%, 75/233) (OR 14.09, 95% CI [2.12, 93.48], *p* = 0.006).

**Conclusion:**

A positive MRI T2-weighted image of the lumbar disc with HIZ indicates disc degeneration. In addition, HIZ may be a specific indicator for the physical diagnosis of discogenic low back pain. A more advanced degree of disc degeneration on the basis of HIZ positivity corresponded to a greater probability of discography-induced consistent pain, whereas the degree of disc degeneration on the basis of HIZ negativity was less correlated with contrast-induced consistent pain.

## Introduction

Discogenic low back pain (DLBP) refers to all degenerative diseases of the lumbar intervertebral discs that do not have nerve tissue compression (excluding lumbar disc herniation and lumbar spinal stenosis, among other factors) as the main manifestation [1]. Crock [2] first suggested in 1970 that abnormalities in the internal structure and metabolic function of the intervertebral disc could cause low back pain, which was further described in 1986 [3] as intervertebral disc disruption (IDD), which has also been interpreted as internal disc derangement. In 1987, Milette [4] suggested that low back pain due to disruption of the nucleus pulposus within the disc and the rupture of the annulus fibrosus (which occurs when the nucleus pulposus leaks into the ruptured annulus fibrosus fissure without apparent herniation of the annulus fibrosus) be referred to as DLBP. The introduction of this concept has led to a deeper understanding of low back pain and is of great significance in guiding clinical diagnosis and treatment. At present, the pathogenesis of DLBP has not been fully elucidated, and there is still a lack of specific methods for diagnosis. Discography is currently recognized as being the gold standard for the diagnosis of DLBP and can clarify the identity of the responsible disc. According to the International Academy of Pain Classification, the criteria for diagnosing discogenic pain should include the concept that painful symptoms should be induced by discography and that the diseased disc should be detectable on CT scan. Additionally, as a control, there should be at least one disc that cannot induce painful symptoms in response to the same stimulus. According to this standard, discography requires a control negative disc, which requires at least one normal discography as a negative disc control, thus indirectly creating a disruption to the negative disc and likely inducing a negative disc herniation. Therefore, as an invasive procedure, disc pin puncture has the potential to cause and accelerate damage to the annulus fibrosus and nucleus pulposus, which may accelerate lumbar disc degeneration. There are still some clinical methods in use for the diagnosis of lumbar DLBP, such as intradiscal block, McKenzie mechanics diagnostic and therapeutic techniques, and spinous process oscillatory stimulation, which have some value in the diagnosis of DLBP. When considering the possible errors caused by the leakage of local anesthetic, more trials are needed to validate the diagnosis of DLBP via intradiscal blocks [5]. Therefore, effective methods and tests that are applied to the diagnosis of DLBP are a hot topic of concern for most clinical practitioners.

The high intensity zone (HIZ) on MRI of the lumbar spine was first reported by Aprill [6] in 1992 and refers to a small, independent, and confined high signal zone that is located at the posterior edge of the fibrous ring on T2-weighted images of the lumbar spine, which is separated from the nucleus pulposus but has a higher signal than the nucleus pulposus. Via clinical studies, Aprill demonstrated that HIZ can induce pain in approximately 90% of cases when the contrast agent spills during discography due to the rupture of the annulus fibrosus, and this pain replicates the patient's usual lower back pain symptoms (known as the pain replication), thus suggesting that HIZ is an important sign for the diagnosis of painful disc rupture. Since Aprill’s discovery of the HIZ, many scholars have studied the area surrounding the HIZ via comparisons of MRI and discography, and their understanding of the HIZ has not been consistent; moreover, there have been debates about its role and significance. Some scholars consider the HIZ as an imaging marker for disc fibrous annulus tears and discogenic lower back pain [7–10]. The presence of HIZ in a single-segment disc with posterior annulus fibrosus on MRI can more reliably indicate that the disc is the source of pain, and neither low disc signal nor HIZ changes can 95% exclude the disc as the source of pain [11]. Furthermore, HIZ has been shown to predict pain with a sensitivity of 26.7%, a specificity of 95.2%, a negative predictive value of 47%, and a positive predictive value of 88.9% [12]. However, some other scholars disagree with the abovementioned viewpoint and believe that the actual diagnostic value of HIZ for discogenic lower back pain is limited [13, 14]. HIZ was also present in 24% of asymptomatic individuals, and 40% of discograms without HIZ were positive [15]. Moreover, HIZ can worsen, decrease, or even disappear over time, and 40.6% of cases exhibit no change [16], with little correlation observed with clinical symptoms [17].

In response to the controversy of HIZ in the diagnosis of DLBP, two meta-analyses with similar content were published by Zang [18] in 2014 and Fang [19] in 2017 to evaluate the correlation between HIZ and the gold standard interstitial discography in the diagnosis of DLBP. It was concluded that the presence of lumbar disc MRI HIZ predicted abnormal disc morphology, as well as the fact that there was a significant correlation between positive HIZ and discography pain replication and that HIZ could be used as a valid indicator for clinically responsible gap determination and diagnosis of DLBP. However, the number of included articles in the 2 studies was small, and the methodology of each included study had varying degrees of limitations; thus, the included studies may have been subject to selection bias and implementation bias. To further improve the reliability of the meta-analysis results and to provide more detailed and credible evidence-based medical evidence, we conducted the present study to increase the number of relevant research papers in recent years and to continuously update high-quality papers. Therefore, this study investigated the relationship between HIZ and positive lumbar discography through meta-analysis, thus providing a basis for the study of the mechanism of correlation between MRI high signal area and DLBP and providing evidence-based medical evidence for clinical purposes.

## Materials and methods

### Literature inclusion criteria

This meta-analysis was performed according to the Cochrane Collaboration Center [20] and MOOSE (Meta-analysis of Observational Studies in Epidemiology) [21] methodological guidelines. The selected literature represented clinical observational studies in which patients with low back pain underwent MRI (including sagittal T2WI scans) and discography of the lumbar spine and correlated the HIZ observed on MRI with positive discography, morphological changes in the disc, and pain replication. There were no gender or age restrictions on patients; moreover, there were no restrictions on the time of publication.

### Retrieval strategy

The PubMed, EMBASE, Cochrane Library, Science Direct, China Knowledge Network, Wanfang Database, and China Biomedical Literature databases were searched from approximately January 1992 to August 2023.The search terms included discogenic low back pain, DLBP, low back pain, HIZ or high intensity-zone(s), MRI and / or discography. Electronic searches were supplemented with manual searches of reference lists of all retrieved review articles, primary studies, and abstracts from meetings to identify other studies not found in the electronic searches. The literature was searched by two authors (L. Yang and WH. Li) independently.

### Literature quality evaluation

The evaluation of the literature was independently performed by two authors by using the Strengthening the Reporting of Observational Studies in Epidemiology Statement (STROBE) [22] observational study evaluation criteria. The quality of the literature was assessed on the following 3 levels: Grade A, wherein the literature meets more than 80% of the STROBE criteria; Grade B, wherein the literature meets 50–80% of the STROBE criteria; and Grade C, wherein the literature meets less than 50% of the STROBE criteria. The title and abstract of each paper were independently read by two researchers to select suitable studies according to the inclusion criteria, and any studies that may be included in the meta-analysis were read in full and translated (if necessary). In cases of disagreement between two researchers on the literature assessment, the decision was resolved through discussion and negotiation or third-party arbitration.

### Extraction and analysis of data

Data from the included literature on the correlation of HIZ with abnormal disc morphology and pain replication outcomes were first extracted. Meta-analysis of the extracted data was performed by using Revman 5.1.1 software provided by the Cochrane Collaboration Network. The *X*^2^ test was first used to determine the heterogeneity of the clinical trial results. If *p* < 0.1 and *I*^2^ > 50%, there was considerable heterogeneity among the included studies. For those results with heterogeneity, the causes of heterogeneity were first analyzed and treated with a sensitivity analysis, and for those in which statistical heterogeneity could not be eliminated in the literature (and if they were clinically consistent), they were combined and analyzed with a random-effects model. Without heterogeneous literature data, a fixed effects model was chosen. The odds ratio (OR) and 95% confidence interval (CI) were calculated for the count data. In addition, for the measurement data, the mean difference (MD) and its 95% CI were calculated when the same scale was used to assess the same efficacy index in each clinical trial. If the meta-analysis showed statistically significant differences, funnel plots or loss-of-safety factors were used to analyze the publication bias.

## Results

### Literature search results and evaluation of methodological quality

**Fig. 1:**
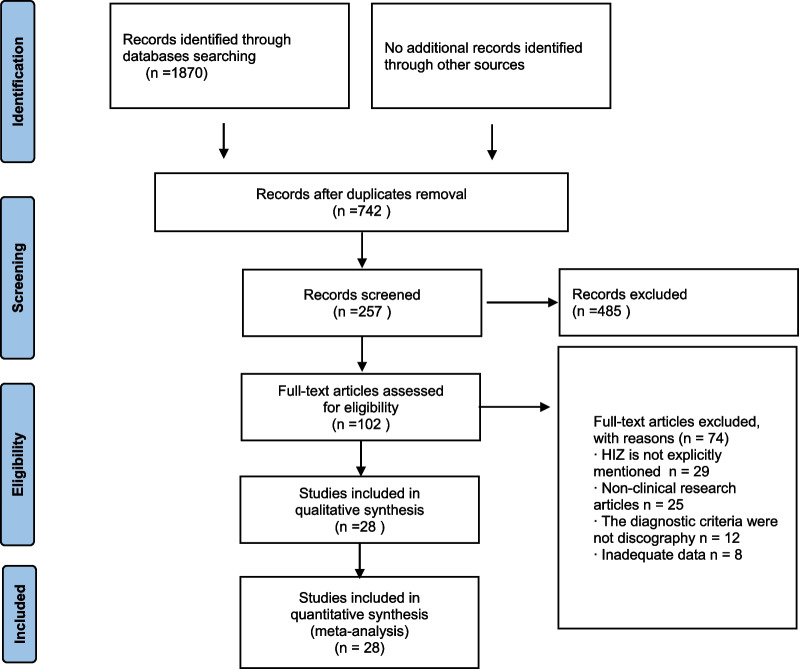
Flow diagram of the study selection process

After searching the literature, 28 papers that met the inclusion criteria were finally included [6, 8, 9, 12, 15, 23–45] for meta-analysis, as shown in Fig. 1. These studies were published in English and Chinese. All of the 28 papers were observational studies investigating the correlation between the HIZ phenomenon in lumbar disc MRI and disc degeneration and imaging findings. According to the literature quality evaluation criteria, 18 papers were graded A, and 10 papers were graded B. The results are shown in Table 1.

**Table 1:** Characteristics and qualities of studies included in the meta-analysis

Author	Country	Study design	Magnetic field intensity	Average age	Male	Female	Described discography method	Is the statistical method correct?	Methodologic quality
Aprill [6]	Australia	CS	0.6 T	No stata	357	143	Yes	Yes	A
Ricketson [23]	America	CCT	No stata	40.9	17	12	Yes	Yes	B
Schellhas [8]	America	RA	1.5 T	37.5	No stata	No stata	Yes	Yes	A
Saifuddin [12]	UK	RA	0.5–1.5 T	42	31	27	Yes	Yes	A
Smith [24]	America	RA	1.5 T	46	36	36	Yes	Yes	A
Ito [25]	America	CCT	1.5 T	37	17	22	Yes	Yes	A
Carragee [15]	America	RA	1.5 T	36.4	25	17	Yes	Yes	B
Lam [9]	UK	RA	1.5 T	42	52	21	Yes	Yes	A
Lim [26]	Korea	CCT	1.5 T	43	20	27	Yes	Yes	A
Kang [43]	Korea	RA	1.5 T	46	No stata	No stata	Yes	Yes	A
Chen [28]	China	CCT	1.5 T	40.11	64	29	Yes	Yes	B
López [29]	Germany	CCT	No stata	43.2	10	21	Yes	Yes	B
Wang [31]	China	CCT	No stata	37.8	26	11	Yes	Yes	A
Chelala [30]	America	CCT	No stata	43	367	338	Yes	Yes	B
Guo [33]	China	CCT	No stata	No stata	25	30	Yes	Yes	B
Ma [34]	China	CCT	1.5 T	37.8	18	25	Yes	Yes	B
Li [35]	China	CCT	1.5 T	51.2	40	26	Yes	Yes	A
Peng [36]	China	CCT	No stata	41	146	61	Yes	Yes	B
Liu [37]	China	CCT	1.5 T	39.3	52	24	Yes	Yes	A
Liu [38]	China	CCT	1.5 T	22.3	39	15	Yes	Yes	A
Liu [39]	China	CCT	1.0 T	51.36	48	34	Yes	Yes	B
Liu [40]	China	CCT	1.5 T	45.3	30	20	Yes	Yes	A
Qiu [41]	China	CCT	1.5 T	38.9	20	28	Yes	Yes	A
Cui [42]	China	CCT	No stata	35.6	15	7	Yes	Yes	B
Peng [27]	China	CCT	1.5 T	38.8	39	13	Yes	Yes	A
Wang [32]	China	CCT	1.5 T	49.4	347	290	Yes	Yes	A
Liu [44]	China	CCT	3.0 T	40.5	44	58	Yes	Yes	A
Bartynski [45]	America	RA	1.5 T	43.1	19	25	Yes	Yes	A

CS, cohort study; RA, retrospective analysis; CCT, case–control study

### Results of the meta-analysis

#### Relationship between HIZ and the morphology of the imaging disc

**Fig. 2:**
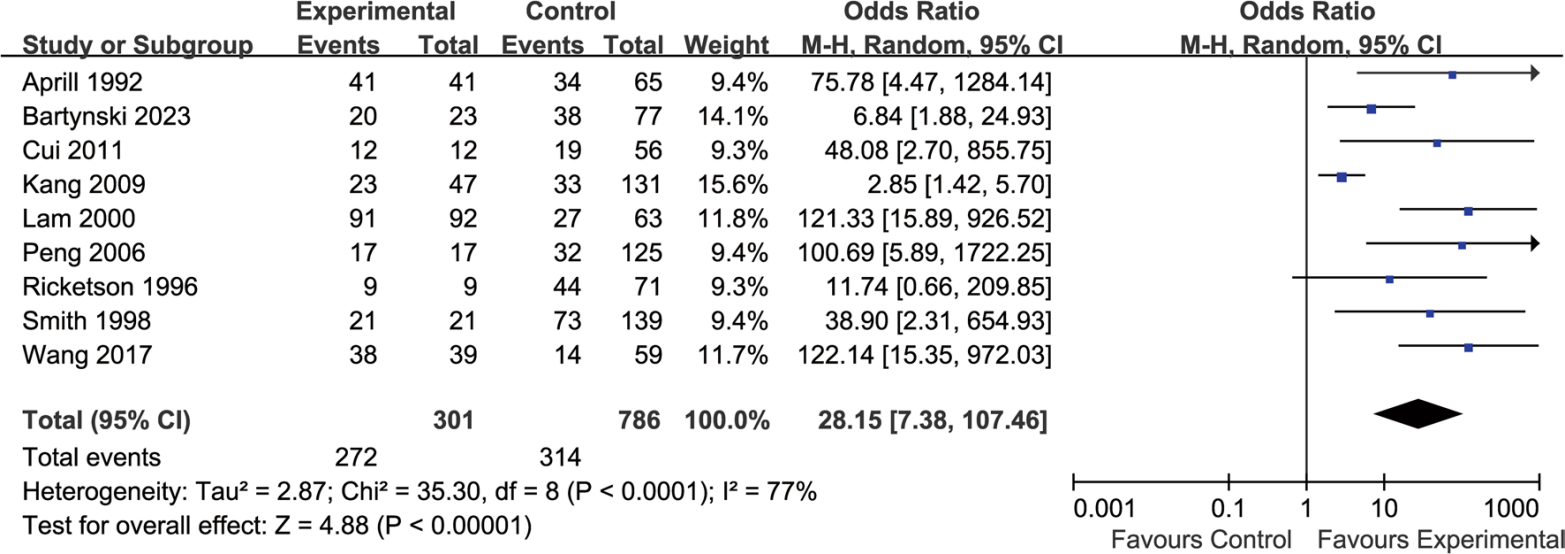
Relationship between HIZ and radiographic disc morphology

According to the 1987 Dallas discography grading system [46], discs of grade 3 or higher are considered to be abnormal. Nine publications [6, 9, 23, 24, 27, 31, 42, 43, 45] have observed the relationship between the presence or absence of HIZ and morphological irregularities in discography (Fig. 2). There was statistical heterogeneity observed across the literature (*p* < 0.00001, *I*^2^ = 77%), and a random-effects model was used, with OR chosen as the indicator for the combined analysis of effect sizes. A total of 301 cases of HIZ-positive discs were included in the literature, including 272 cases with degeneration at grade 3 or higher; moreover, 786cases of HIZ-negative discs were included, including 314 cases with degeneration at grade 3 or higher. The results suggest a statistically significant correlation between positive HIZ and abnormal disc morphology in discography (OR 28.15,95% CI [7.38107.46], *p* < 0.00001).

#### Relationship between HIZ and discography pain replication

**Fig. 3:**
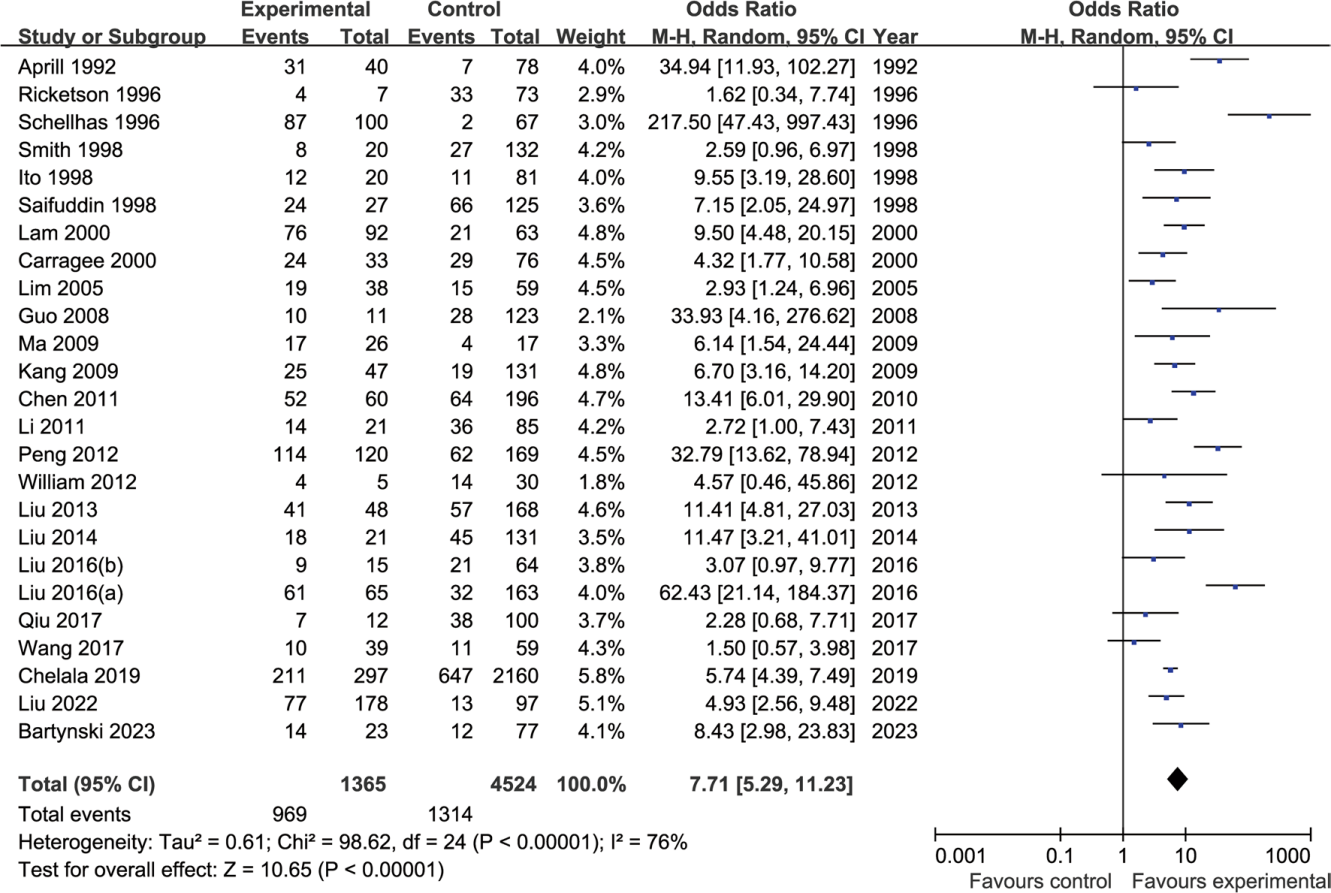
Relationship between HIZ and pain replication in discography

Twenty-five publications [6, 8, 9, 12, 15, 23–26, 28–31, 33–41, 43–45] examined the relationship between the presence or absence of HIZ and discography pain replication (Fig. 3). There was statistical heterogeneity observed across the literature (*p* < 0.00001, *I*^2^ = 76%), and a random-effects model was used, with OR chosen as the indicator for the combined analysis of effect sizes. HIZ-positive discs were included in 1365 cases, of which 969 were contrast-induced painful discs, whereas 4524 HIZ-negative discs were included, of which 1314 were contrast-induced painful discs. The results suggested a higher incidence of HIZ-positive induced consistent pain during lumbar discography (71.0%, 969/1365), with a statistically significant difference compared with HIZ-negative (29.0%, 1314/4524) (OR 7.71, 95% CI [5.29, 11.23], *p* < 0.00001).

#### Relationship between HIZ and painful replication of intervertebral discs with abnormal contrast morphology

**Fig. 4:**
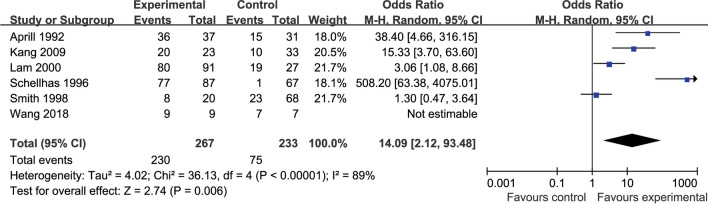
Relationship between HIZ and painful replication of disc with abnormal angiographic morphology

Six publications [6, 8, 9, 24, 32, 43] have observed the relationship between the production of consistent pain and the presence or absence of HIZ in morphologically abnormal discs (grade 3 or higher) (Fig. 4). There was statistical heterogeneity observed across the literature (*p* < 0.00001, *I*^2^ = 89%), and a random-effects model was used, with OR chosen as the indicator for the combined analysis of effect sizes. The results included 267 cases of HIZ-positive with grade 3 or higher degenerated discs, of which 230 cases induced consistent pain, as well as 233 cases of HIZ-negative with grade 3 or higher degenerated discs, of which 75 cases induced consistent pain. The results suggested a high incidence of lumbar disc degeneration grade 3 or higher with HIZ-positive discography-induced consistent pain (86.1%, 230/267), with a statistically significant difference compared with the incidence of HIZ-negative (32.2%, 75/233) (OR 14.09, 95% CI [2.12, 93.48], *p* = 0.006).

## Discussion

Our findings showed that the relationship between the presence or absence of HIZ and abnormal disc morphology on discography was observed in nine of the included papers, with statistical heterogeneity observed between the papers (*I*^2^ = 77%). HIZ-positive discs were included in 301cases, including 272cases with degeneration at grade 3 or higher, with an incidence of 90.1%; in addition, HIZ-negative discs were included in 786 cases, including 314 cases with degeneration at grade 3 or higher, with an incidence of 39.9%. The results suggested a statistically significant correlation between a positive HIZ and abnormal disc morphology in discography (*p* < 0.00001). Five of these papers [23, 27, 31, 42, 45] were exactly graded according to the Dallas discography grading system’ moreover, 2 papers [6, 24] improved on the Dallas classification, and 2 papers [9, 43] did not use Dallas grading, but the articles depicted interdiscal degeneration in detail.

The relationship between the presence or absence of HIZ and positive lumbar discograms was observed in 25 of the included studies, and there was statistical heterogeneity observed among the studies (*I*^2^ = 76%), which was mainly due to the low quality of the included studies and the small sample size. HIZ-positive discs were included in 1365 cases, of which 969 cases were contrast-induced painful discs, with an incidence of 71.0%, whereas 4524 cases with HIZ-negative discs were included; among them, 1314 cases of contrast-induced painful interstitial discs occurred, with an incidence of 29.0%. The results suggested a statistically significant higher incidence of HIZ-positive induced consistent pain during lumbar discography (*p* < 0.00001). Six of the included papers examined the relationship between HIZ positivity with disc degeneration grade 3 or higher and discography-induced consistent pain. There was statistical heterogeneity observed across the literature (*I*^2^ = 89%), which was mainly due to the risk of bias in the included literature and the small sample size. HIZ positivity with grade 3 or higher degenerated discs was included in 267 cases, of which 230 cases induced consistent pain, and HIZ negativity with grade 3 or higher degenerated discs was included in 233 cases, of which 75 cases induced consistent pain. The results suggested a high incidence of lumbar disc degeneration grade 3 or higher with HIZ-positive discography-induced consistent pain (86.1%), with a statistically significant difference compared with the incidence of HIZ-negative (32.2%) (*p* = 0.006).

The pathological mechanism of HIZ has not been fully elucidated, and most scholars believe that the appearance of HIZ is associated with inflammation of the fibrous ring and tearing of the fibrous ring with neovascular granulation tissue. Anatomical studies have revealed that the fissures of each fibrous ring injury are filled with a mucus-like substance that showed a high signal on T2-weighted MRI images, and the presence of this substance was presumed to represent an inflammatory response [47]. Further MRI-enhanced scans with Gd-DTPA as a contrast agent demonstrated that the viscoelastic material in these damaged fissures was inflammatory granulation tissue [48]. The expression levels of tumor necrosis factor (TNF-*α*) and macrophages (CD68) were significantly higher in the fibrous rings in the region where the HIZ was located than in the fibrous ring tissue adjacent to the HIZ and in normal controls [49, 50]. Histological examinations of the HIZ-containing fibrous ring specimen demonstrated that the normal lamellar structure of the fibrous ring in the area of the HIZ was lost and replaced by small disordered chondrocytes, fibroblasts, and neovascular tissue that extended from the inner medulla to the outer layer of the fibrous ring. Vascular proliferation and inflammatory cell infiltration from the edge of the fibrous ring injury zone to the middle and interior of the nucleus pulposus were observed, and these neovascularized tissues interconnect multiple damaged areas of the annulus fibrosus, or they are distributed in the inner 1/3 of the fibrous ring at the junction with the nucleus pulposus. Moreover, the laminar structure of the fiber ring adjacent to the HIZ also gradually changes from relatively ordered to disordered, whereas the laminar structure of the fiber ring away from the HIZ is relatively normal [27]. The morphology and location of the HIZ and the annular fissure connected to the radial rupture in the lumbar intervertebral disc were observed to be consistent by using stereolocation combined with CT discography; thus, the presence of the HIZ was considered to suggest the formation of an annular fissure in the fibrous annulus [51, 52]. These studies suggest that the HIZ represents an annular fissure connected to a radial rupture of the fibrous ring and that its main pathological substance is inflammatory granulation tissue in the fissure of the fibrous ring injury and the foci of calcification or ossification of the fibrous ring.

As early as 1986, Crock et al. [3] proposed the theory of "internal rupture of the intervertebral disc". They suggested that after the rupture of the annulus fibrosus, the exposed nucleus pulposus could induce macrophage infiltration, which could subsequently release large amounts of inflammatory mediators, such as interleukins (ILs) and tumor necrosis factors (TNF-α). These inflammatory mediators can enhance phospholipase A2 (PLA2) activity by promoting the expression of nitric oxide (NO) and prostaglandin E2 (PGE2), which enhances and prolongs the nociceptive effect of histamine, 5-hydroxytryptamine, bradykinin, and other nociceptive factors on nerve endings that grow into the disc along the fissure of the annulus fibrosus. This theory suggests that the inflammatory response and nerve fibers growing into the disc are the main pathological basis of DLBP, and many scholars have studied the mechanism of HIZ-induced low back pain from this perspective.

To explore the inflammatory mechanisms underlying HIZ as a specific imaging marker of painful intervertebral discs, Ren et al. [49] found that a large number of proliferating chondrocytes and vascular endothelial cells were observed in the fibrous rings in the area of HIZ, and the expression levels of TNF-α and CD68 immunopositive cells were significantly higher than those in the surrounding fibrous rings, whereas the controls exhibited little or no expression. The widely distributed area of granulation tissue strips within the HIZ is the site of origin of discography pain and DLBP [53]. Additionally, the presence of HIZ was significantly correlated with the grade of fibrous ring rupture. A higher degree of rupture corresponded to a higher percentage of high-signal areas appearing on MRI [54]. Therefore, the inward growth of nerve fibers induced by the inflammatory response may be the main mechanism of HIZ-induced low back pain.

The abovementioned study shows that the degree of disc fibrous ring rupture is positively correlated with HIZ, and the presence of HIZ suggests a high likelihood of vertebral fibrous ring rupture; moreover, the main pathological parenchyma associated with HIZ may be inflammatory granulation tissue in the fissure of the fibrous ring injury and foci of calcification or ossification of the fibrous ring. This effect is consistent with our meta-analysis, which demonstrated a90.1% incidence of HIZ-positive disc degeneration grade 3 or higher and 39.9%of HIZ-negative disc degeneration grade 3 or higher. Furthermore, there was a significant difference between the two conditions (*p* < 0.00001), thus suggesting that most of the HIZ-positive intercalated discs had ruptured fibrous rings. Our meta-analysis showed that the rate of HIZ-positive discography-induced consistent pain was 71.0%, and the rate of HIZ-negative discography-induced consistent pain was 29.0%. The difference was significant (*p* < 0.00001), thus suggesting a significant correlation between HIZ positivity and DLBP symptoms, which is consistent with related studies. The results of our meta-analysis showed that the incidence of consistent pain in HIZ-positive patients with disc degeneration grade 3 or higher was 86.1%, and the incidence of consistent pain in HIZ-negative patients with disc degeneration grade 3 or higher was 32.2%, with significant differences observed between the two conditions, thus suggesting that a higher degree of disc degeneration corresponded to a greater possibility of consistent pain in HIZ-positive patients. Compared with contrast-induced consistent pain without considering the degree of disc degeneration, the incidence of HIZ-positive with grade 3 or higher degeneration consistent pain was 15.1% higher, whereas the incidence of HIZ-negative with grade 3 or higher disc degeneration consistent pain was only 3.2% higher. This result suggests that HIZ-negative-induced consistent pain does not correlate well with the degree of disc degeneration.

In summary, we believe that a positive T2-weighted image of the lumbar disc on MRI with HIZ indicates disc degeneration, and most of the degeneration is greater than grade 3. Approximately 70%of patients with positive HIZ on MRI T2-weighted images of lumbar discs may have discography-consistent pain; therefore, HIZ may be a specific indicator for the physical diagnosis of DLBP. Moreover, a greater degree of disc degeneration on the basis of positive HIZ indicated a greater probability of discography-induced consistent pain, whereas the degree of disc degeneration on the basis of negative HIZ is less correlated with contrast-induced consistent pain.

## Data Availability

All the datasets were available from Dr. Lei Yang upon reasonable request.
